# Supplementary material for: Plasma proteomic profiles predict individual future health risk
Source: Nat Commun. 2023 Nov 28;14:7817. doi: 10.1038/s41467-023-43575-7 (PMC10684756; doi:10.1038/s41467-023-43575-7)
Supplement: Supplementary file 1 — Supplementary Information [file 41467_2023_43575_MOESM1_ESM.pdf]

Supplementary Information

Supplementary Fig. 1: Flowchart of leave-one-region-out cross-validation pipeline

Supplementary Fig. 2: Proteomic neural network

Supplementary Fig. 3: Scatterplot of ProRS percentile versus observed event rates

Supplementary Fig. 4: Kaplan-Meier survival plots of stratified ProRS tertiles

Supplementary Fig. 5: Calibration plots of CPH fitted ProRS and clinical predictor sets

Supplementary Fig. 6: Calibration plots of CPH fitted ProRS and clinical predictor sets

Supplementary Fig. 7: Net benefit curves of CPH fitted ProRS and clinical predictor sets

Supplementary Fig. 8: Net benefit curves of CPH fitted ProRS and clinical predictor sets

Supplementary Fig. 9: Fitted scatterplot of comorbid network

Supplementary Fig. 1: Flowchart of leave-one-region-out cross-validation pipeline

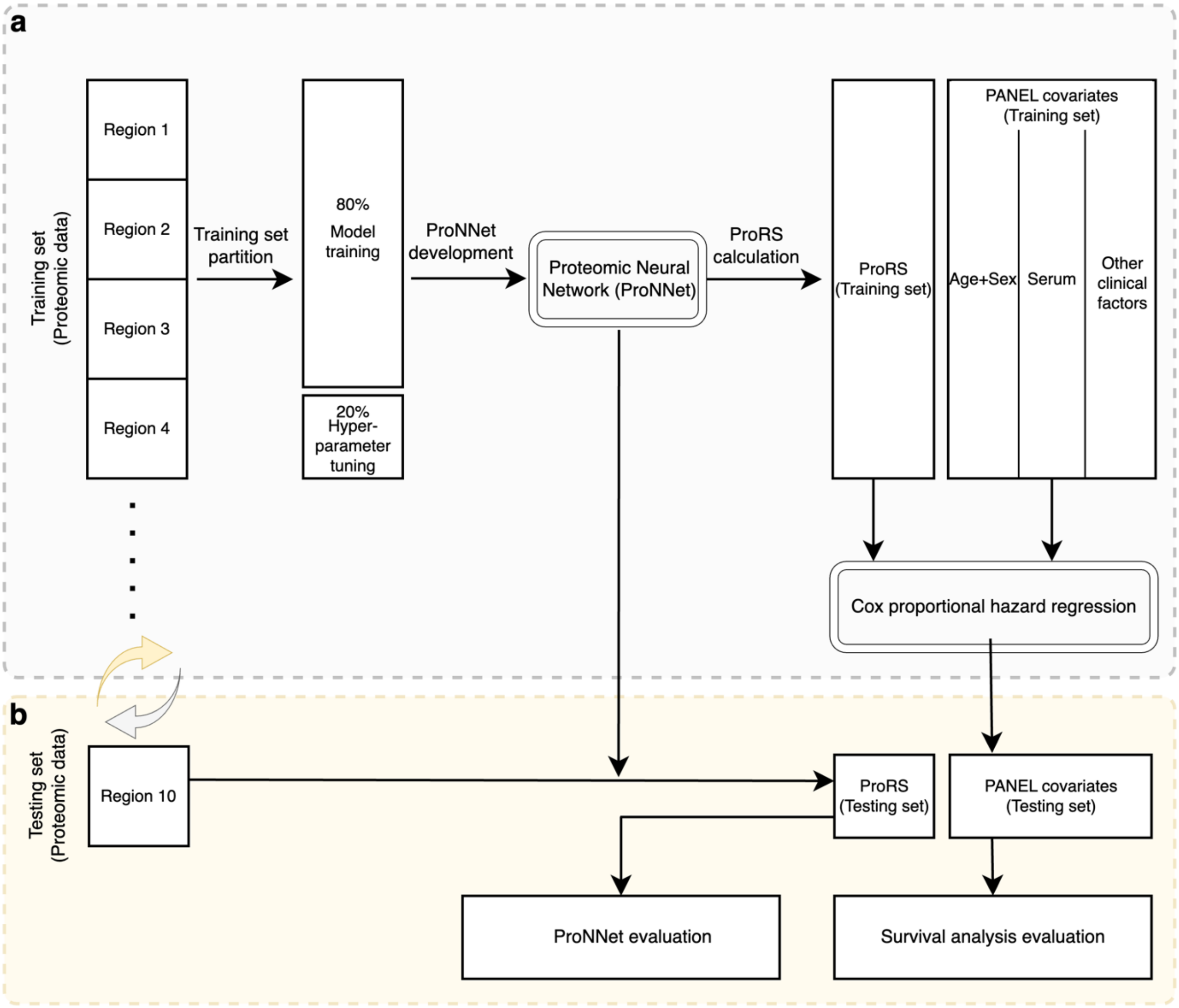

Regions were partitioned based on geographical locations of participants 'assessment centers. A demo partition in the figure leveraging region 1 to region 9 as training set and the remaining region 10 as testing set, this partition iterative repeated until all folds of data has been used as both training and testing sets.

a. Model development based on temporal training data.

b. Model evaluation based on temporal testing data.

Supplementary Fig. 2: Proteomic neural network

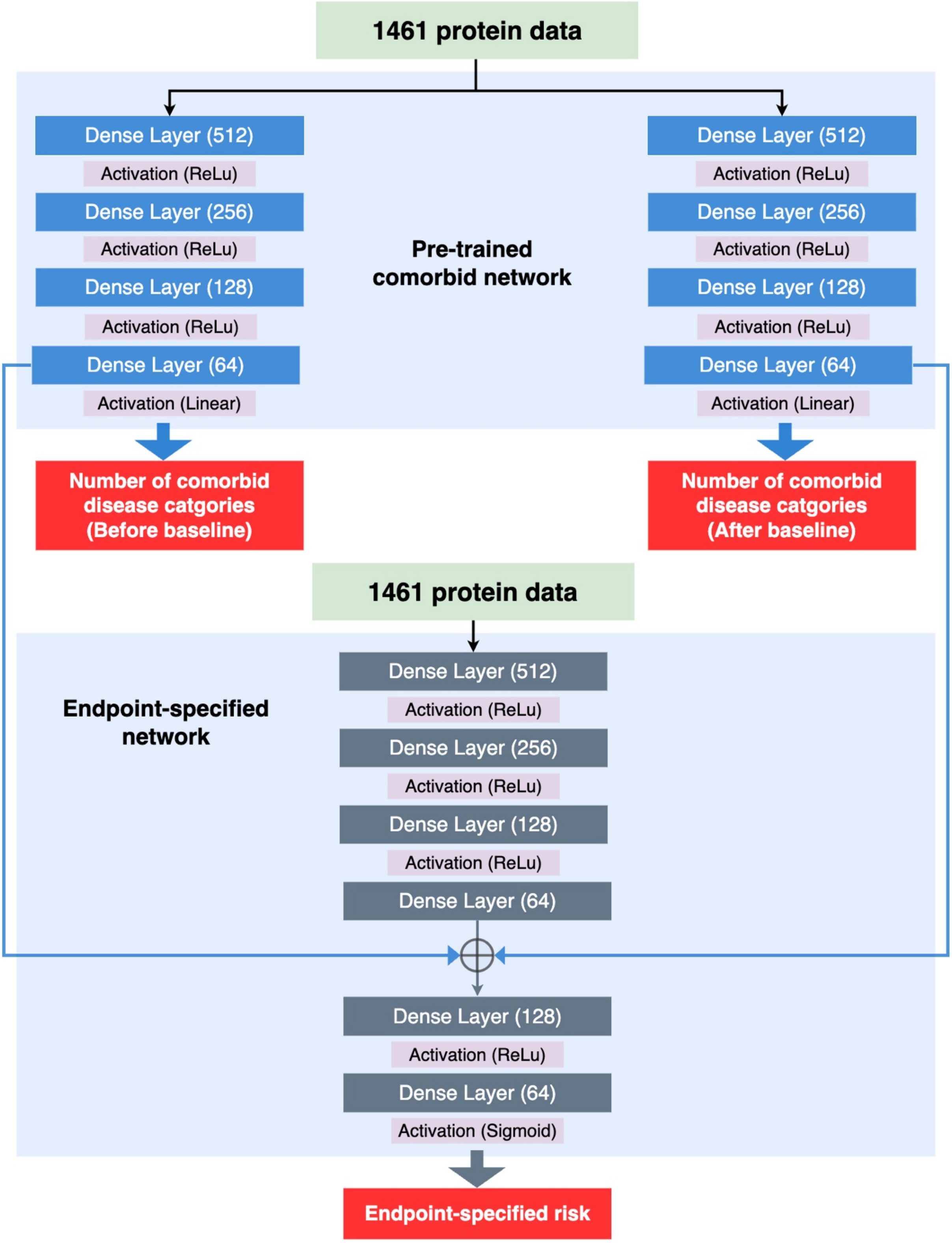

The pre-trained weights of comorbid network were shared by all 45 endpoints, and there were 45 endpoint-specified networks that specifically trained to optimize the prediction on each endpoint.

Supplementary Fig. 3: Scatterplot of ProRS percentile versus observed event rates

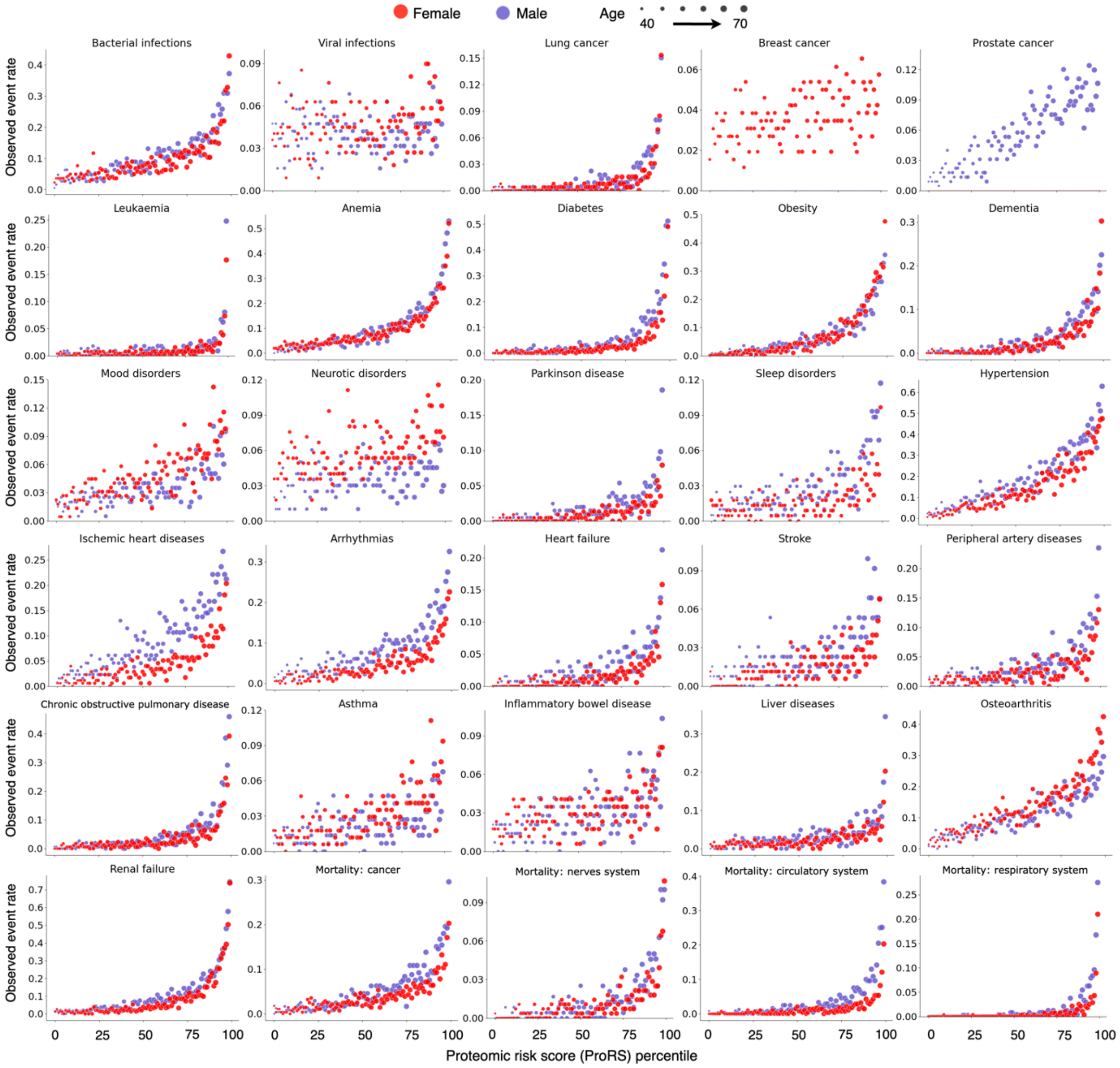

This is a supplementary figure to main Figure 2a of anther 30 endpoints.

Supplementary Fig. 4: Kaplan-Meier survival plots of stratified ProRS tertiles

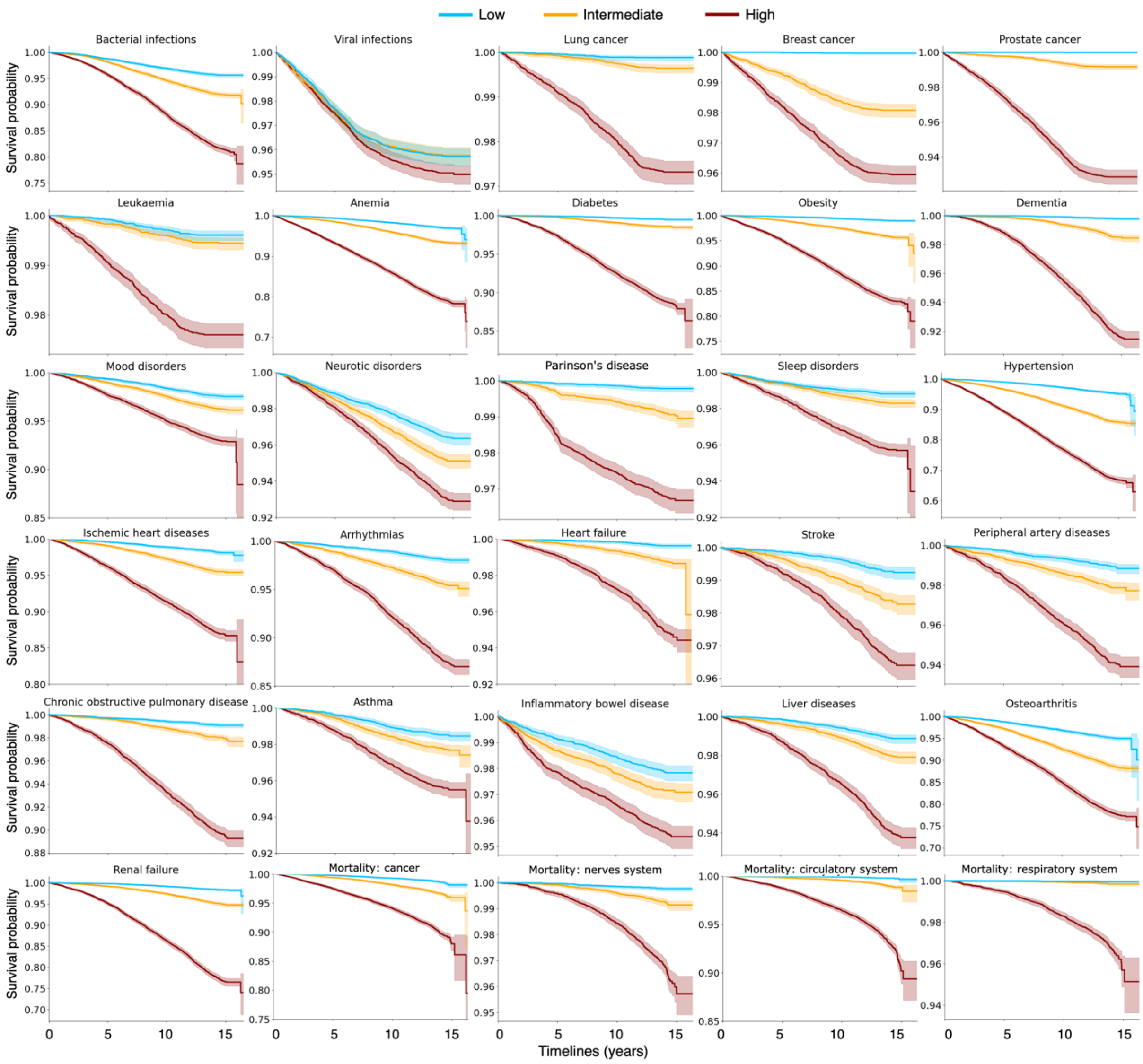

This is a supplementary figure to main Figure 2b of anther 30 endpoints.

Supplementary Fig. 5: Calibration plots of CPH fitted ProRS and clinical predictor sets

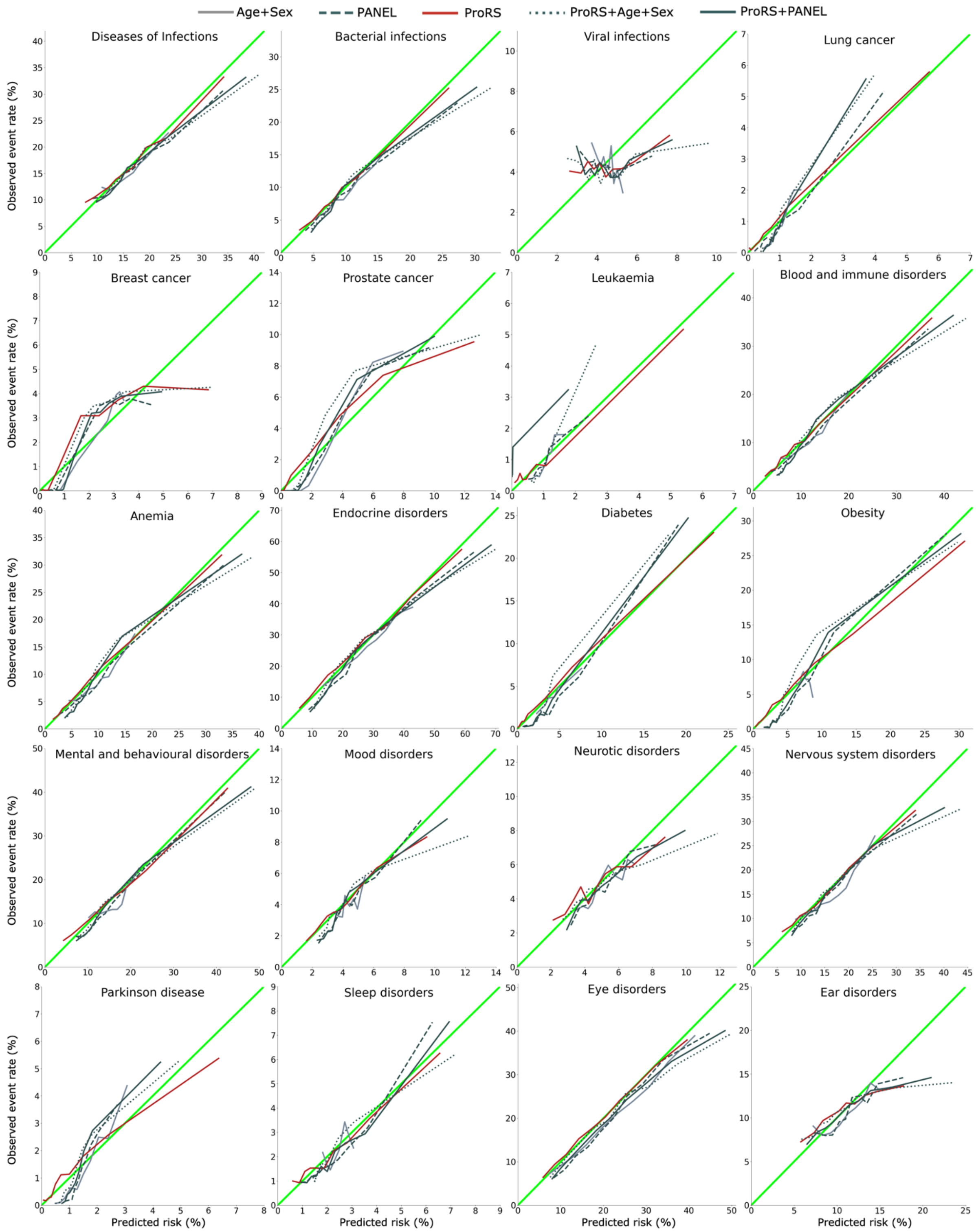

This is a supplementary figure to main Figure 6a-d of anther 41 endpoints.

Supplementary Fig. 6: Calibration plots of CPH fitted ProRS and clinical predictor sets

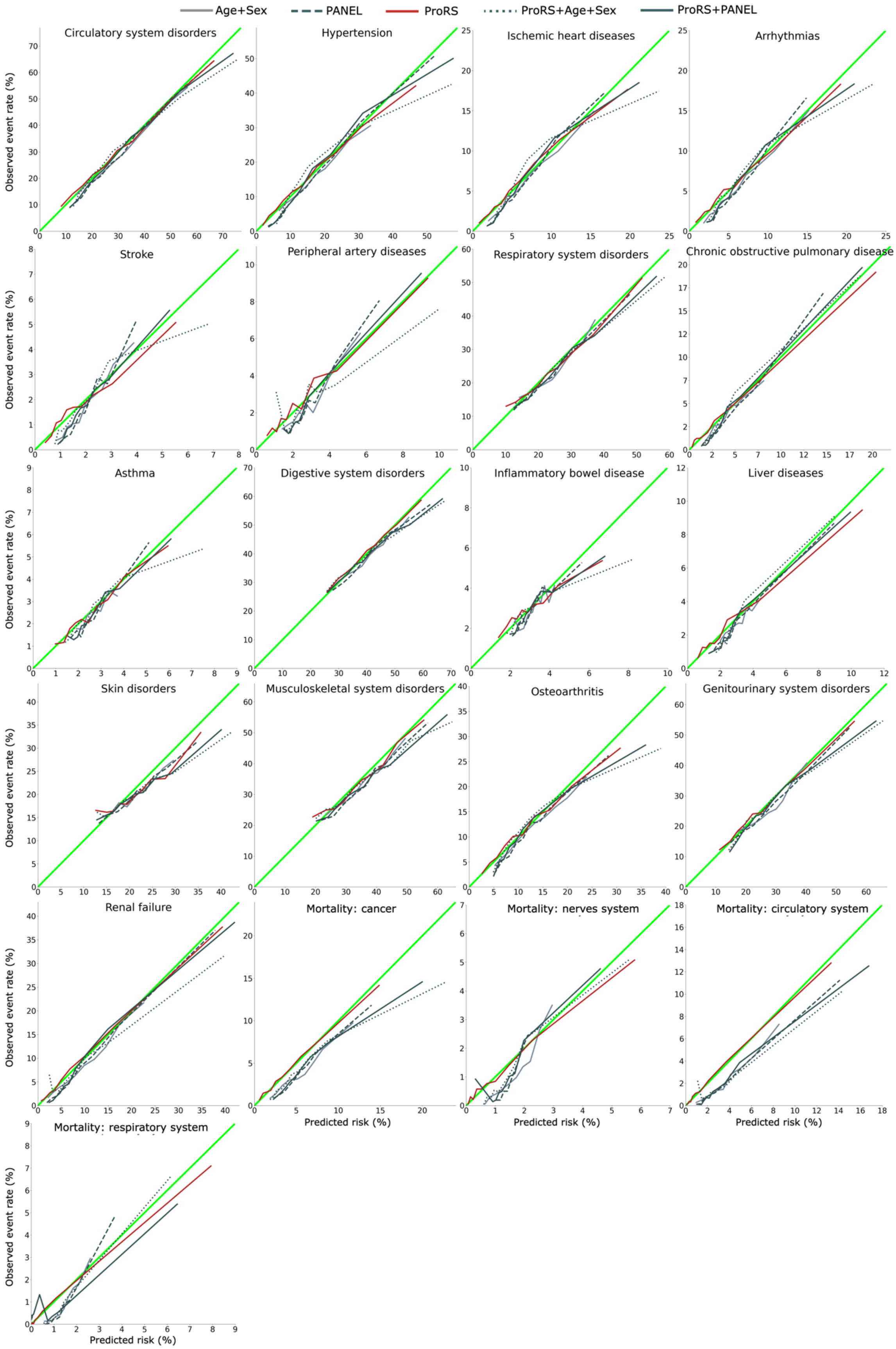

This is a supplementary figure to main Figure 6a-d of anther 41 endpoints.

Supplementary Fig. 7: Net benefit curves of CPH fitted ProRS and clinical predictor sets

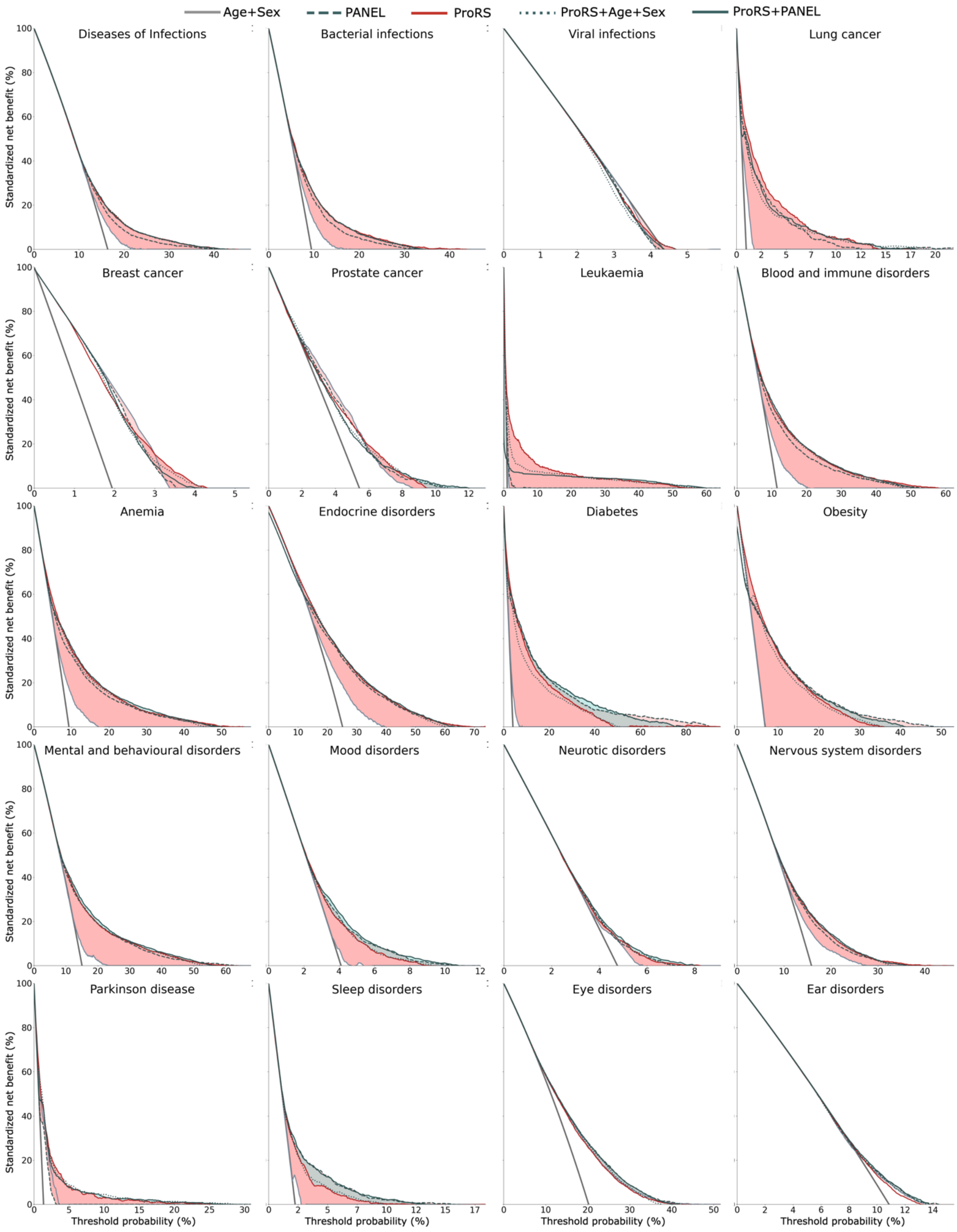

This is a supplementary figure to main Figure 6e-h of anther 41 endpoints.

Supplementary Fig. 8: Net benefit curves of CPH fitted ProRS and clinical predictor sets

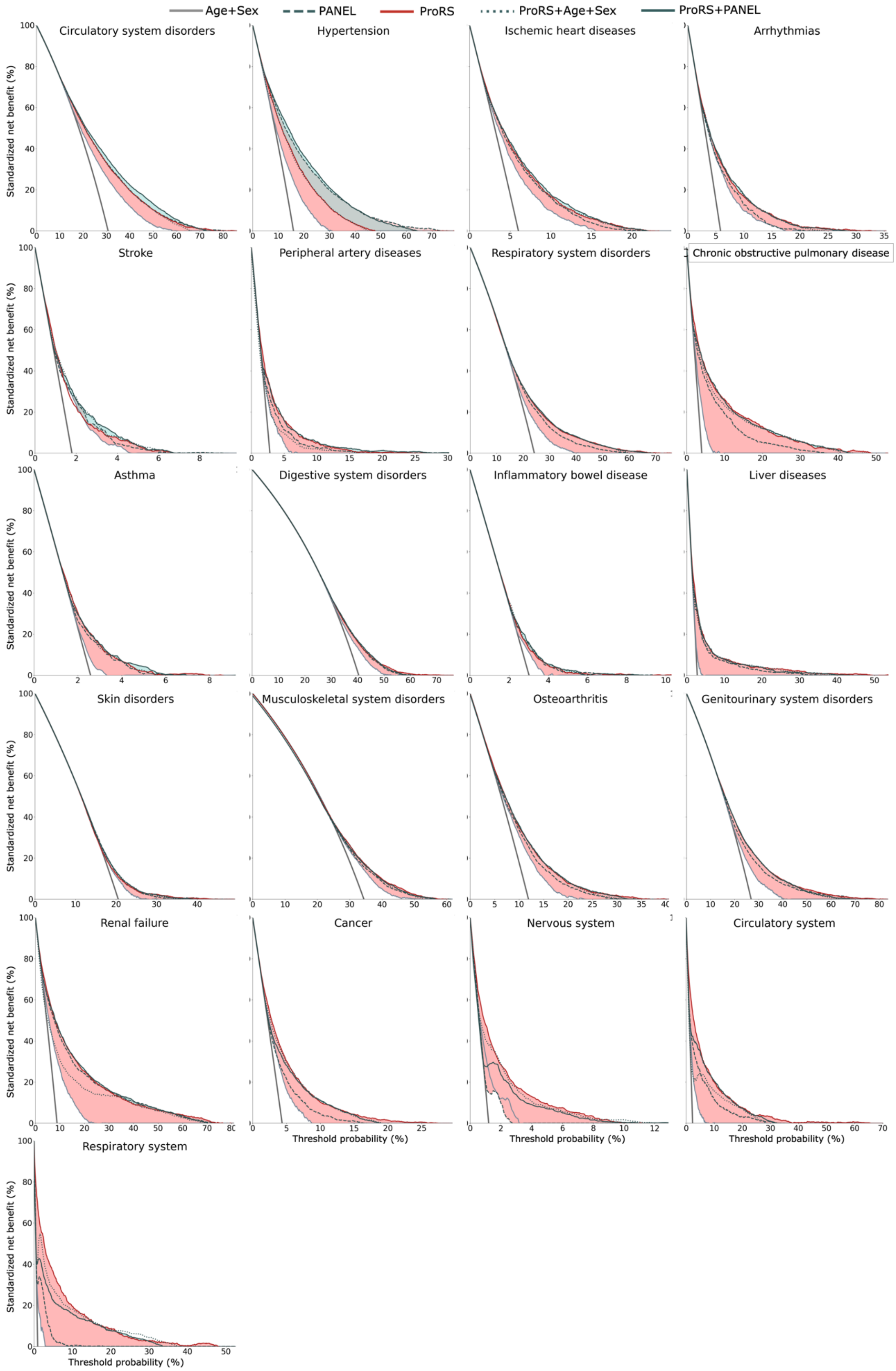

This is a supplementary figure to main Figure 6e-h of anther 41 endpoints.

Supplementary Fig. 9: Fitted scatterplot of comorbid network

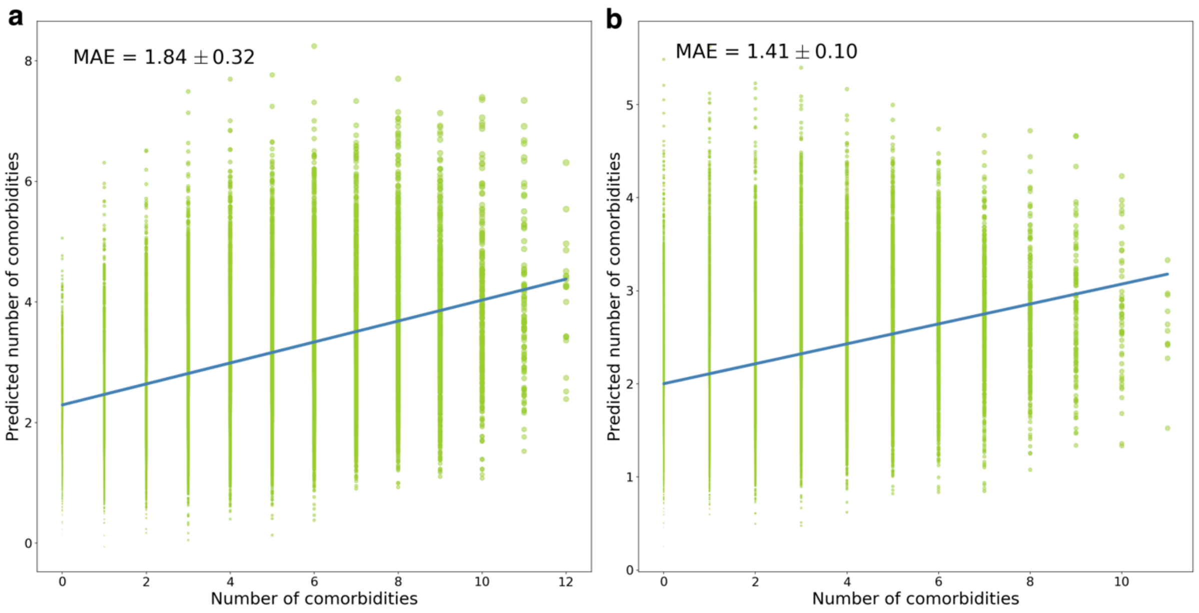

Number of comorbidities was defined as the summed number of 14 disease categories listed in ICD-10 code.

a. Predicted versus actual number of comorbidities before baseline.

b. Predicted versus actual number of comorbidities after baseline.
