# Supplementary material for: Plasma proteomic profiles predict individual future health risk
Source: Nat Commun. 2023 Nov 28;14:7817. doi: 10.1038/s41467-023-43575-7 (PMC10684756; doi:10.1038/s41467-023-43575-7)
Supplement: Supplementary file 3 — Description of Additional Supplementary Files [file 41467_2023_43575_MOESM3_ESM.pdf]

Title: Supplementary Data 1.
Description: Endpoint read codes, incident events, exclusions
Exclusion criteria to each endpoint was defined as any baseline or self-reported events of its disease category. Excluded participants were not included in further analysis.

Title: Supplementary Data 2.
Description: Summary statistics of clinical predictors
Continuous data are described as median [interquartile range], and categorical variables are presented as numbers (percentages).
Abbreviation: ML, machine learning; KNN, K-nearest neighbours

Title: Supplementary Data 3.
Description: Participants statistics of geographical data partitions
Data were partitioned into ten folds based on geographical locations of 22 assessment centers in the UK. Participants were reported with number and its proportion over the whole cohort (n=52006). Female, male and death were reported with number and the proportion within its site.

Title: Supplementary Data 4:
Description: Relationship between ProRS and individuals' age and sex
Pearson's correlation was used to calculate corrlations between individual's age and ProRS. Student t-test was used to test the difference of ProRS between males and females. P-value was derived under two-sided tests. Median and IQR reported for ProRS times 100. Correlation and summary statistics of ProRS were reported from aggregated data from cross-validation.
Abbreviation: CI, confidence interval; IQR, interquartile range; ProRS, proteomic risk score

Title: Supplementary Data 5:
Description: Risk stratification of ProRS tertile groups
The table reports the proportion (95% confidence interval) of endpoint incidence under tertle groups of ProRS; odds ratio was calculated based top tertile over bottom tertile. All metrics were reported with mean and 95% confidence interval derived from cross-validation.
Abbreviation: CI, confidence interval

Title: Supplementary Data 6:
Description: Predictive performance of ProRS across different incident time-windows
The proteomic risk score (ProRS) was initially derived based on all incidents per endpoint, and it was then employed to test its predictive performance under different incident time-windows. Metrics of accuracy, sensitivity, specificity were derived based on the cut-off upon achievement of the largest Youden index (=sensitivity+specificity-1). All metrics were reported with mean and 95% confidence interval derived from cross-validation.
Abbreviation: AUC, area under Receiver-Operating-Characteristic (ROC) curve; APR, area under Precision-Recall curve

Title: Supplementary Data 7:
Description: C-index of different combinations of ProRS with multiple covariate sets
For three covariate sets of Age+Sex, Serum and PANEL, two models were performed: covariate set aloine (only) and combined with ProRS (+ProRS). C-index statistics were reported with mean and 95% confidence interval derived from cross-validation. P-values were calculated through R package *compareC* (V1.3.2) based on aggregated predicted probabilities across all cv-folds and calculated from two-sided tests; significance are marked as: *: <0.05; **: <0.01; ***: <0.001. direction indicator represents the direction of C-index between two compared predictions, e.g. ProRS versus covariates or ProRS+covariates versus covariates. Abbreviation: ProRS, proteomic risk score

Title: Supplementary Data 8:
Description: Hazard ratios of ProRS adjusted under multiple covariate sets
Serum included 25 covariates derived from serum; PANEL contained all 54 clinical predictors, see ST2 for list of predictors. Proteomic risk score (ProRS) were normalized when fitting the Cox model. Hazard ratios and confidence intervals were derived based on aggregated data from cross-validation. Corresponding p-values were derived under two-sided tests.
Abbreviation: CI, confidence interval, CPH, Cox proportional hazard regression, HR, hazard ratio

Title: Supplementary Data 9:
Description: Endpoint-specific top-15 (1%) proteins ranked based on SHAP values
The top-15 (1%) proteins were sorted and selected based on SHAP values derived from the endpoint-specific machine learning classifier. Values in the () represent the shap values after dividing the summation of all 1461 proteins.

Title: Supplementary Data 10:
Description: 1461 plasma proteins adopted in the study
