# Supplementary material for: Plasma proteomic profiles predict individual future health risk
Source: Nat Commun. 2023 Nov 28;14:7817. doi: 10.1038/s41467-023-43575-7 (PMC10684756; doi:10.1038/s41467-023-43575-7)
Supplement: Supplementary file 14 — Reporting Summary [file 41467_2023_43575_MOESM14_ESM.pdf]

nature portfolio

Corresponding author(s): DARP; NCOMMS-23-26906A

Last updated by author(s): Oct 11, 2023

Reporting Summary

Nature Portfolio wishes to improve the reproducibility of the work that we publish. This form provides structure for consistency and transparency in reporting. For further information on Nature Portfolio policies, see our Editorial Policies and the Editorial Policy Checklist.

Statistics

For all statistical analyses, confirm that the following items are present in the figure legend, table legend, main text, or Methods section.

n/a	Confirmed	
☐	☒	The exact sample size (*n*) for each experimental group/condition, given as a discrete number and unit of measurement
☐	☒	A statement on whether measurements were taken from distinct samples or whether the same sample was measured repeatedly
☐	☒	The statistical test(s) used AND whether they are one- or two-sided<br>*Only common tests should be described solely by name; describe more complex techniques in the Methods section.*
☐	☒	A description of all covariates tested
☐	☒	A description of any assumptions or corrections, such as tests of normality and adjustment for multiple comparisons
☐	☒	A full description of the statistical parameters including central tendency (e.g. means) or other basic estimates (e.g. regression coefficient) AND variation (e.g. standard deviation) or associated estimates of uncertainty (e.g. confidence intervals)
☐	☒	For null hypothesis testing, the test statistic (e.g. *F*, *t*, *r*) with confidence intervals, effect sizes, degrees of freedom and *P* value noted<br>*Give P values as exact values whenever suitable.*
☒	☐	For Bayesian analysis, information on the choice of priors and Markov chain Monte Carlo settings
☒	☐	For hierarchical and complex designs, identification of the appropriate level for tests and full reporting of outcomes
☒	☐	Estimates of effect sizes (e.g. Cohen's *d*, Pearson's *r*), indicating how they were calculated

Our web collection on statistics for biologists contains articles on many of the points above.

Software and code

Policy information about availability of computer code

Data collection	No software used during data collection, and data can be directly download after approval of access application.
Data analysis	Machine learning model of ProNNet was developed through in Keras (v2.7.0) under Python 3.9. Survival analysis of CPH model was implemented with CoxPHFitter from the lifelines package (v0.27.4) under Python. Data imputation and statistical analysis was conducted through Python package of scikit-learn (v1.2.2). Statistical comparison between paired C-index was implemented through R package Compare C. The code used in this study can be accessed at https://github.com/jasonHKU0907/FutureHealthProteomicPrediction.

For manuscripts utilizing custom algorithms or software that are central to the research but not yet described in published literature, software must be made available to editors and reviewers. We strongly encourage code deposition in a community repository (e.g. GitHub). See the Nature Portfolio guidelines for submitting code & software for further information.

Data

Policy information about availability of data

All manuscripts must include a data availability statement. This statement should provide the following information, where applicable:

- Accession codes, unique identifiers, or web links for publicly available datasets
- A description of any restrictions on data availability
- For clinical datasets or third party data, please ensure that the statement adheres to our policy

The data used in the present study are available from UKB with restrictions applied. Data were used under license and are thus not publicly available. Access to the UKB data can be requested through a standard protocol (https://www.ukbiobank.ac.uk/register-apply/). Data used in this study are available in the UK Biobank

under application number 19542.

Research involving human participants, their data, or biological material

Policy information about studies with human participants or human data. See also policy information about sex, gender (identity/presentation), and sexual orientation and race, ethnicity and racism.

Reporting on sex and gender	Sex (biological) has been included in our analysis which was self-reported during UK Biobank recruitment. Gender has not been collected and thus does not appear in our analysis.
Reporting on race, ethnicity, or other socially relevant groupings	Ethnicity has been included in our analysis which was self-reported during UK Biobank recruitment. Social economic status was measured using Townsend deprivation index calculated immediately prior to participant joining UK Biobank. It was calculated based on the preceding national census output areas. Each participant is assigned a score corresponding to the output area in which their postcode is located. No information of sex and ethnicity was involved in the calculation of social economic status. The ethnicity and social economic status was leveraged as covariates in our analysis as has been previously reported as important risk factors to specific diseases and been adopted to established clinical risk scales.
Population characteristics	This study adopted 52,006 participants with plasma proteomics data available in the UK Biobank, and the population had a median age of 58 years (interquartile range (IQR) 50-64), of whom 53.9% were female, and mainly consisted of white ethnicity (93.7%). Median years of education were 11 (IQR 10-15), body mass index was 26.8 (IQR 24.2-29.9), systolic blood pressure was 138.0 mmHg (IQR 126.0-152.0), and 5481 (10.6%) people were current smokers. During a median follow-up time of 14.1 (IQR 13.4-14.8) years until March 2023, 5,625 participants died (10.82%), 7,654 people developed cancer (15.76%), and the most common specific diseases were hypertension (n=4,911, 15.96%) and anemia (n=4,528, 9.31%).
Recruitment	The UKB enrolled the participants aged 40-69 years between 2006 and 2010 for baseline assessments in 22 centers across the UK. The assessment visits included comprehensive range of data collections, covering interviews, physical measures, biological samples, imaging, and genotyping. The database is linked to national health datasets, including primary care, hospital inpatient, death, and cancer registration data.
Ethics oversight	UK Biobank has received ethical approval from the North West Multi-centre Research Ethics Committee (MREC, https://www.ukbiobank.ac.uk/learn-more-about-uk-biobank/about-us/ethics), and informed consent through electronic signature was obtained from study participants. This study utilized the UK Biobank Resource under application number 19542.

Note that full information on the approval of the study protocol must also be provided in the manuscript.

Field-specific reporting

Please select the one below that is the best fit for your research. If you are not sure, read the appropriate sections before making your selection.

- [x] Life sciences
- [ ] Behavioural & social sciences
- [ ] Ecological, evolutionary & environmental sciences

For a reference copy of the document with all sections, see nature.com/documents/nr-reporting-summary-flat.pdf

Life sciences study design

All studies must disclose on these points even when the disclosure is negative.

Sample size	The UK Biobank encompasses data of over 500,000 participants, from whom, 52,705 have proteomics data available were included. As the study focuses on proteomic data modeling, those who had over 30% of missingness in proteomic measurements were excluded, and finally 52,006 were left for analysis. Sample size are sufficient with this study on simple statistical tests and machine learning models as our sample sizes are larger than previous studies performed in relevant research fields.
Data exclusions	Participants who had over 30% of missingness in proteomic measurements were excluded. For modeling and analysis of each endpoint, any participants indexed from self-reported clinical records or any incidents indexed before the baseline of the respective disease category were excluded. For analysis of sex-specified disease, e.g. breast cancer and prostate cancer, counterpart sex participants were excluded.
Replication	The study was implemented extensively through an leave-one-region-out cross-validation to replicate and confirm the findings.
Randomization	The avoid overfitting and explore the generalizability of the developed machine learning models, this study adopted a leave-one-region-out cross-validation strategy for model development and evaluation. The study cohort was split based on the geographical locations of total 22 assessment centers, and they were merged into ten regions in the UK as our data partition criteria (see Supplementary Table 3 for detailed population characteristics within each region). The model was developed using data from nine regions and evaluated in the remaining region, and such a scheme iteratively looped until all regions have been used for both model development and model validation.
Blinding	This study did not require blinding as no subjective evaluation by on observer was involved.

Reporting for specific materials, systems and methods

We require information from authors about some types of materials, experimental systems and methods used in many studies. Here, indicate whether each material, system or method listed is relevant to your study. If you are not sure if a list item applies to your research, read the appropriate section before selecting a response.

Materials & experimental systems

n/a	Involved in the study
☒	☐ Antibodies
☒	☐ Eukaryotic cell lines
☒	☐ Palaeontology and archaeology
☒	☐ Animals and other organisms
☒	☐ Clinical data
☒	☐ Dual use research of concern
☒	☐ Plants

Methods

n/a	Involved in the study
☒	☐ ChIP-seq
☒	☐ Flow cytometry
☒	☐ MRI-based neuroimaging

Plants

Seed stocks	*Report on the source of all seed stocks or other plant material used. If applicable, state the seed stock centre and catalogue number. If plant specimens were collected from the field, describe the collection location, date and sampling procedures.*
Novel plant genotypes	*Describe the methods by which all novel plant genotypes were produced. This includes those generated by transgenic approaches, gene editing, chemical/radiation-based mutagenesis and hybridization. For transgenic lines, describe the transformation method, the number of independent lines analyzed and the generation upon which experiments were performed. For gene-edited lines, describe the editor used, the endogenous sequence targeted for editing, the targeting guide RNA sequence (if applicable) and how the editor was applied.*
Authentication	*Describe any authentication procedures for each seed stock used or novel genotype generated. Describe any experiments used to assess the effect of a mutation and, where applicable, how potential secondary effects (e.g. second site T-DNA insertions, mosiacism, off-target gene editing) were examined.*
