# Supplementary material for: Plasma proteomic profiles predict individual future health risk
Source: Nat Commun. 2023 Nov 28;14:7817. doi: 10.1038/s41467-023-43575-7 (PMC10684756; doi:10.1038/s41467-023-43575-7)
Supplement: Supplementary file 15 — Source Data [file 41467_2023_43575_MOESM15_ESM.zip › SourceData/Source Data Description.docx]

Descriptions of Source Data used to plot figures in the manuscript of

Plasma proteomic profiles predict individual future health risk

**Figure2_SourceData (folder):** 15 Excel files are provided containing data of 15 endpoints (14 disease categories and all-cause mortality) to plot the frequency plots (Figure 2a) and Kaplan-Meier survival plots (Figure 2b). It provides data of *ProRS* (Proteomic Risk Score), *incident event* (1 for incident event; 0 for no incident event), *censored_years* (censor time), *Age* (participants’ age, it was normalized for anonymized purpose), *Sex* (0 for female; 1 for male).

**Figure3_SourceData.xlsx:** Data to plot Figure 3. It contains the predictive performance and corresponding confidence intervals of 45 endpoints modelled using different predictor sets.

**Figure4_SourceData.xlsx:** Data to plot Figure 4. It contains the top selected proteins and the corresponding SHAP values of 15 endpoints (14 disease categories and all-cause mortality). It provides the data of *Pro_code* (protein code), *ShapValue* (SHAP value of each protein when modelling on different endpoint), *InTop15* (1 if the protein was ranked among the top 1%; 0 otherwise), *ImpSum* (summed SHAP values of each protein), *NbTop15* (how many times the protein was ranked among the top 1% for each of the 15 endpoints), *DiseaseCode* (disease codes).

**Figure5_SourceData.xlsx:** Two tabs are provided, Figure5a and Figure5b to plot SHAP value of cancer (Figure 5a) and dementia (Figure 5b), respectively. The SHAP value of 1,000 participants were provided for each of the 1,461 proteins. It provides the data of *shap_values* (SHAP value of corresponding proteins), *Pro_values* (protein values, normalized for encryption purpose), *Pro_code* (proteion code), *TopPro* (1 if the protein was ranked among the top 1%; 0 otherwise).

**Figure6_SourceData.xlsx:** Four tabs are provided, Figure6a&e and Figure6b&f, Figure6d&h were used to plot calibration and net benefit plots of cancer, dementia, heart failure and all-cause mortality, respectively. It provides the data of *incident_event* (1 for incident event; 0 for no incident event), *risk_ProRS* (predicted risks derived using ProRS), *risk_AgeSex* (predicted risks derived using age and sex), *risk_PANEL* (predicted risks derived using PANEL predictors), *risk_ProRS_AgeSex* (predicted risks derived using age, sex and ProRS), *risk_ProRS_Panel* (predicted risks derived using PANEL predictors and ProRS).
